# Current evidence for the medical treatments of systemic juvenile idiopathic arthritis

**DOI:** 10.1186/1546-0096-12-S1-I9

**Published:** 2014-09-17

**Authors:** Nicolino Ruperto

**Affiliations:** 1 Pediatria II, Reumatologia, PRINTO, Istituto G. Gaslini, Genova, Italy

## 

Systemic juvenile idiopathic arthritis (JIA) is the most severe category within the group of arthritis which are classified under the umbrella term of JIA. Systemic JIA has been considered a therapeutic orphan until few years ago when the disease was treated primarily with corticosteroids with the known side effect especially on child growth. More recently the availability of new treatment modalities with biologic agents such as anti L6 and anti IL1 therapies have greatly advanced the possibilities for these children to be adequately treated.

This lecture will describe the current status of the treatment for systemic JIA and the future perspectives.

## Disclosure of interest

N. Ruperto Grant / Research Support from: The Gaslini Hospital, which is the public Hospital where I work as full time public employee, has received contributions from the following industries: Abbott, BMS, "Francesco Angelini", GlaxoSmithKline (GSK), Hoffman-La Roche, Italfarmaco, Janssen, Novartis, Pfizer, Sanofi Aventis, Schwarz Biosciences, Sobi, Xoma, Wyeth, Speakers Bureau of: Abbott/AbbVie, Astellas, Alter, AstraZeneca, Boehringer, BMS, CD-Pharma, Celgene, Crescendo Bio, EMD Serono, Hoffman-La Roche, Italfarmaco, Janssen, MedImmune, Medac, Novartis, Novo Nordisk, Pfizer, Sanofi Aventis, Vertex Pharmaceuticals, Servier

